# Treatment of Compound Fractures of Skull, with Report of Case

**Published:** 1895-03

**Authors:** William S. Goldsmith

**Affiliations:** Atlanta, Ga., Lecturer on Genito-Urinary and Rectal Diseases, Assistant to Chair of Surgery Atlanta Medical College; 241-243 Equitable


### TREATMENT OF COMPOUND FRACTURE OF SKULL, WITH REPORT OF CASE.

BY WM. S. GOLDSMITH, M. D., ATLANTA, GA.,
Lecturer on Genito-Urinary and Rectal Diseases, Assistant to Chair of Surgery Atlanta Medical College.

The subject of this paper was suggested by the treatment of the case which I shall describe, and is not intended as an exhaustive *resume* of the literature on fractures of this variety. The diagnosis and treatment of fractures of the vault of the skull are of such importance and interest that it is with reluctance that I confine myself to the case in point, and the remarks I may make incident to this variety of injuries.

On the afternoon of December 21, 1893, I was called to see J. K., brick mason, age 30, living a short distance from the city, who, no doubt, at the suggestion of the approaching holidays, decided to begin the festivities by firing off an old musket, which, as was subsequently learned, had hung on a rack for quite a year, its disuse occasioned by the fact of its being a family relic. The gun exploding, a piece of the breech pin, half an inch in diameter and two inches long, with a shoulder one-half inch from the screw end, struck him full in the forehead and this screw portion, one-half inch in length, was embedded in the skull, two inches above the superciliary ridge and one and a half inches to the left of the frontal suture.

I saw him two hours after the accident, lying on the floor, with head elevated, perfectly rational, but frightened nearly out of his wits by the crowd of anxious neighbors.

The missile was so firmly fixed in the skull that it was with considerable difficulty that I effected its removal, without further injury to the bone or soft parts. Just below this aperture in the skull was a gash one and a half inches in length. There was also a small lesion to the left of the wound which afterwards proved to be the entrance of a shot which was found half-embedded in the skull and was easily removed.

At the time of my first examination I found his pulse 86, pupils normal, temperature not taken. Nearly the whole of the upper part of his face and ears were powder-burned and stained. Putting him to bed and preparatory to the further application of the dressings, I gave him a ¼ grain of morphine with $\frac{1}{150}$ grain atropia, hypodermically.

Upon the removal of the foreign body, a clot of brain substance and blood gushed out, followed by about half a teaspoonful of brain substance mingled with blood, I irrigated the cavity with a 1 to 3,000 bichloride solution, washing away a quantity of debris, and after thoroughly cleansing the surrounding skin and hair, applied a dressing of bichloride gauze, cotton and bandage. I left ¼ grain morphine powders with his nurse, with instructions to administer at intervals of five hours until I called the next morning.

I saw him the next day at noon, resting quietly, but with temperature of 100$\frac{3}{5}$ pulse 56, respiration 14. As soon as I approached the bed I detected a peculiar odor which indicated an immediate change of dressings, and when removed I found small particles of disintegrated brain tissue clinging to that portion which covered the wound. I then irrigated the cavity with the bichloride solution, breaking down and washing away a quantity of decomposing brain tissue which has a peculiarly characteristic odor. I dressed the wound as before and advised his family of the gravity of the symptoms and suggested operation, which they willingly consented to. Detained at my office much longer than I anticipated, I at last reached his house at 9 o'clock that night, and with the assistance of my friends, Drs. Westmoreland and Gill, the latter administering the ether, proceeded with the operation. At that time pulse 54, but good; temperature 100$\frac{1}{5}$ respiration improved. His head shaved, scalp cleaned with ether and washed with a 1 to 1,000 bichloride solution and all antiseptic precaution observed, considering our surroundings. I made crucial incision, the lines of incision, three inches in length, crossing at the right border of the fracture. Dissecting the tissues and periosteum entire, and tying a silk thread at the point of each flap, retracted them. I applied a one inch trephine on the right side of the wound, taking in a small area of the fractured surface. Removing the button of bone I found that we had the superior longitudinal sinus to deal with.

We caught it up with two pair of artery forceps, cutting between them and leaving them on until completion of operation. The opening afforded by the trephine made my field of exploration much clearer, and with my finger and a small pair of dressing forceps, I succeeded in removing a number of pieces of shattered bone, some of which were finally extracted after repeated and prolonged efforts to grasp them. Being thoroughly satisfied that the cavity was as clear as we could possibly make it, I then cleared the edges of both the trephine and puncture openings of all spiculae of bone so there could not be any cause for future trouble. I then lightly packed the cavity with narrow strips of bichloride gauze, then iodoform gauze as an external dressing, cotton and bandage.

During the operation pulse showed decided improvement and continued strong after operation. Patient recovered from influence of the anæsthetic nicely. I saw him the following morning, found him resting well, pulse 50, temperature 99, respiration 16; slight nausea from effect of anæsthetic, otherwise felt as well as ever. The next day found his pulse 54, temperature normal, nausea disappeared, fine appetite. I removed the dressings and found the wound perfectly healthy-looking and with an entire absence of any odor whatever. I again packed the cavity with gauze and dressed as previously.

I continued the use of the gauze drainage until the cavity completely filled out, after which using only the bichloride gauze and the other dressings necessary for the treatment of any scalp wound. His improvement continued very rapidly and in one month's time was able to come to my office, a distance of five miles in a wagon. He is to-day well and strong and pursuing his occupation and feels no inconvenience whatever from either the injury or result of the operation.

Anticipating the question which will naturally arise, and in explanation of the views I now entertain in reference to fractures of the vault of the skull, I will say, that my delay in waiting nearly thirty hours before operating, was due to my inexperience in the treatment of such injuries and to my conservative views concerning immediate exploratory operations before the appearance of symptoms indicating compression or cerebral irritation. In fractures of this character, I believe that immediate operation is imperative, and that delay, even for a few hours, is oftentimes attended by complications, increasing the danger of operative interference. We had here a condition demanding a radical operation for the extraction of the shattered bone already embedded in the brain substance and the removal of the depressed fragments surrounding the aperture.

The consciousness of having cleared the cavity, as far as possible, of all foreign matter and the establishment of thorough drainage, was gratifyingly rewarded by the immediate and continued improvement of my patient. In the treatment of compound fractures where the elevator does not meet the requirements for the relief of the condition, and it is necessary to resort to the use of the trephine, the question as to the danger attached to this method of operative procedure should not deter us for a moment, as since the advent of antisepsis in surgery, the danger of the operation has been reduced to a minimum, the mortality being a little over one per cent.

Even with the observance of rigid antiseptic precautions many of the fatal terminations of operations for the relief of some injury to the skull or brain, has been ascribed to the use of the trephine, when in a great majority of instances the fatality is the result of some pre-existing condition, the responsibility of which may or may not rest on the surgeon. His extreme conservatism in reference to radical operation, or the confusion of symptoms relative to the diagnosis of the injury, governs him in the course he pursues.

Before the days of antiseptic surgery the conversion of a simple into a compound fracture was a procedure approached with much trepidation by the surgeon. Experience taught him that in the treatment of an open wound leading into the cavity of the skull possessed such danger that he was almost necessarily led to practice the expectant treatment in many cases. Now that mild antiseptic solutions of sufficient strength to prevent the infectious process can be introduced into the cavity of the skull and brain with such remarkable results, it is additional evidence of that most brilliant achievement in the history of modern surgery.

Thorough drainage is one of the most important questions to be considered in the treatment of these fractures. The size and character of the injury is to be considered in deciding upon the most efficient procedure to adopt. Taking the case reported, as an example of the worst form of this class of injuries, where thorough drainage was one of the most important factors in its successful termination, the bichloride gauze, and the same is true of mild iodoform gauze, lightly packed to the bottom of the cavity proved to be the ideal method of drainage. The delicate texture and the resiliency of the gauze is especially commended for its complete absorption of the abundant secretions poured out.

Drainage tubes, no matter of what material, are especially antagonistic toward accomplishing as near as possible that condition of rest to the brain which is so essential for the recovery of tone and the development of the process of repair which is very rapid.

The open treatment of the wound of the scalp possesses superior advantages over the method of suturing the flaps after trephine operation. The open treatment also favors more efficient drainage, which fact is especially impressed upon us where there is already a high degree of inflammation present, before any operative measures are taken.

Of course the progress of repair is much slower when the flaps are brought together to take the chances of partially healing by primary union. This minor objection, however, is of little importance when we take into consideration the danger of suppuration, necessitating a re-opening of the wound, and increasing the danger of exciting some cerebral mischief.

*241-243 Equitable.*

